# Spatial Repolarization Heterogeneity Detected by Magnetocardiography Correlates with Cardiac Iron Overload and Adverse Cardiac Events in Beta-Thalassemia Major

**DOI:** 10.1371/journal.pone.0086524

**Published:** 2014-01-27

**Authors:** Chun-An Chen, Meng-Yao Lu, Shinn-Forng Peng, Kai-Hsin Lin, Hsiu-Hao Chang, Yung-Li Yang, Shiann-Tarng Jou, Dong-Tsamn Lin, Yen-Bin Liu, Herng-Er Horng, Hong-Chang Yang, Jou-Kou Wang, Mei-Hwan Wu, Chau-Chung Wu

**Affiliations:** 1 Department of Pediatrics, National Taiwan University Hospital, Taipei, Taiwan; 2 Department of Medical Imaging, National Taiwan University Hospital, Taipei, Taiwan; 3 Department of Internal Medicine, National Taiwan University Hospital, Taipei, Taiwan; 4 Institute of Electro-optical Science and Technology, National Taiwan Normal University, Taipei, Taiwan; 5 Department of Physics, National Taiwan University, Taipei, Taiwan; Temple University, United States of America

## Abstract

**Background:**

Patients with transfusion-dependent beta-thalassemia major (TM) are at risk for myocardial iron overload and cardiac complications. Spatial repolarization heterogeneity is known to be elevated in patients with certain cardiac diseases, but little is known in TM patients. The purpose of this study was to evaluate spatial repolarization heterogeneity in patients with TM, and to investigate the relationships between spatial repolarization heterogeneity, cardiac iron load, and adverse cardiac events.

**Methods and Results:**

Fifty patients with TM and 55 control subjects received 64-channel magnetocardiography (MCG) to determine spatial repolarization heterogeneity, which was evaluated by a smoothness index of QT_c_ (SI-QT_c_), a standard deviation of QT_c_ (SD-QT_c_), and a QT_c_ dispersion. Left ventricular function and myocardial T2* values were assessed by cardiac magnetic resonance. Patients with TM had significantly greater SI-QT_c_, SD-QT_c_, and QT_c_ dispersion compared to the control subjects (all p values<0.001). Spatial repolarization heterogeneity was even more pronounced in patients with significant iron overload (T2*<20 ms, n = 20) compared to those with normal T2* (all p values<0.001). Log_e_ cardiac T2* correlated with SI-QT_c_ (r = −0.609, p<0.001), SD-QT_c_ (r = −0.572, p<0.001), and QT_c_ dispersion (r = −0.622, p<0.001), while all these indices had no relationship with measurements of the left ventricular geometry or function. At the time of study, 10 patients had either heart failure or arrhythmia. All 3 indices of repolarization heterogeneity were related to the presence of adverse cardiac events, with areas under the receiver operating characteristic curves (ranged between 0.79 and 0.86), similar to that of cardiac T2*.

**Conclusions:**

Multichannel MCG demonstrated that patients with TM had increased spatial repolarization heterogeneity, which is related to myocardial iron load and adverse cardiac events.

## Introduction

Thalassemia major (TM) is a single gene disorder, which results in severely impaired synthesis of the globin chain. Adoption of transfusion regimens and iron-chelating therapy has led to great improvement in life expectancy in the majority of affected individuals [1]–[3]. However, cardiac iron overload, resulting in heart failure and arrhythmia, remains the major cause of death [4]. Iron toxicity to cardiomyocytes not only results in impaired contractility [5], [6], but also causes delayed electrical conduction and increased electrophysiological heterogeneities [7]–[9]. Various electrocardiographic parameters have been investigated in patients with TM, including QT_c_ interval, QT dispersion, and QT variability [10]–[12]. However, the clinical implications of these parameters remain unclear.

Magnetocardiography (MCG) is a medical technique that directly measures the weak magnetic field generated by electrical activity in the heart, using a superconducting quantum interference device (SQUID). With the advances in multichannel MCG systems, this technique has been recognized for its outstanding ability to detect coronary artery disease and cardiac allograft vasculopathy after heart transplantation, through the evaluation of various repolarization indices [13]–[18]. Compared to the 12-lead surface electrocardiogram (ECG), more registration sites in multichannel MCG allow not only a more sensitive calculation of the range of QT intervals, but also a detailed examination of the spatial distribution of QT intervals over the heart. Therefore, the purpose of this study was to investigate the applicability of MCG for detecting repolarization heterogeneity in patients with TM. Additionally, we assessed the hypothesis that MCG-derived indices of spatial repolarization heterogeneity are related to cardiac iron load and adverse cardiac events.

## Methods

### Ethics Statement

The study protocol was approved by the institutional review board of the National Taiwan University Hospital, and written informed consent from all participants was obtained. This study conformed to the principles of the Helsinki Declaration.

### Subjects

From June 2008 to January 2010, patients with transfusion-dependent TM, treated at the Pediatric Hematology Department of National Taiwan University Hospital, served as the eligible population of this study. Patients were transfused every 2 to 4 weeks to keep hemoglobin levels above 10 g/dL. Among the 53 patients enrolled, 3 were excluded from further analysis because of the presence of a conduction disturbance in 12-lead surfance ECG (QRS duration >120 ms). Therefore, a total of 50 patients with TM were studied. The control group for MCG consisted of 55 healthy subjects with similar age and sex as the patient group. Control subjects were free from any cardiovascular disorder, and had no conduction disturbance after evaluation by 12-lead surface ECG.

### Acquisition of SQUID MCG

**Figure 1:**
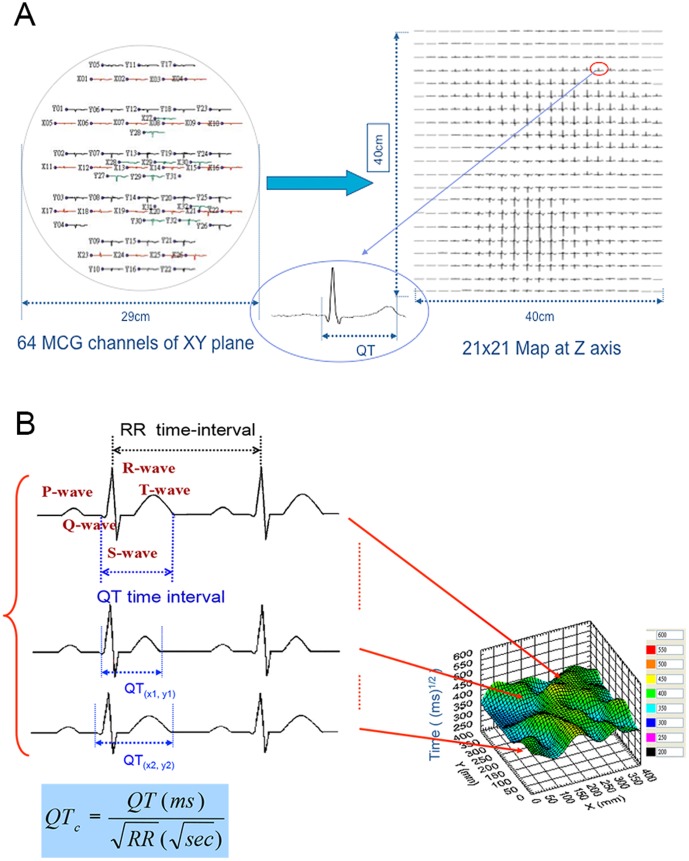
Construction of a QT_c_ contour map from magnetocardiography (MCG). Signals from 64-channel MCG traces before averaging, and the construction of repolarization map with a 21×21 resolution by signal-averaged vector-projected electrocardiogram (A). The spatial distribution of corrected QT (QT_c_) intervals was displayed on a QT_c_ contour map (B).

All participants were assessed by the 64-channel SQUID MCG system (developed by Korea Research Institute of Standards and Science) to detect spatially distributed magnetocardiac signals (Fig. 1A) [19]. To reduce electromagnetic artifacts, the measurements were performed in a magnetically shielded room. The shielding factors of the magnetically shielded room were approximately 60 dB at 1 Hz, 80 dB at 10 Hz, and 100 dB at 100 Hz. Inside this room, the SQUID gradiometers exhibited a noise level of approximately 10 fT/Hz^1/2^ at 100 Hz, and approximately 50 fT/Hz^1/2^ at 1 Hz. The probe was positioned as close to the chest as possible, directly over the heart. MCG recordings were carried out at rest for 100 seconds. Premature beats and the beats just prior to the premature beats were carefully identified and then excluded from analysis. The remaining heart beat signals were averaged, centering on the R-wave peak [13], to obtain time-averaged, one-period magnetocardiac signals for each individual channel over the entire recording period. The QT interval at each channel was measured from the earliest onset of the QRS complex to the latest terminal portion of the T wave based on the time-averaged Bz-t curves by using overlapped MCG waveforms, then visually checked and manually corrected if necessary. The QT interval was then corrected by heart rate (QT_c_), using Bazett's formula (QT_c_ = QT/√R-R, where R-R is the averaged RR interval in seconds). The QT_c_ was used for the construction of the QT_c_ contour map, with a spatial resolution of 21×21 (Fig. 1B). In addition, QT_c_ intervals from all channels were averaged to obtain the mean QT_c_ interval.

### Analysis of Spatial Repolarization Heterogeneity

Based on the QT_c_ contour map, 3 indices were calculated to evaluate spatial repolarization heterogeneity. First, the smoothness index of QT_c_ (SI-QT_c_), modified from previous studies [16], [20], [21], was calculated based on the following formula:
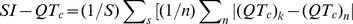
where S is the total number of measured MCG points, Σ_S_ is a summed value of the total measured MCG points, and 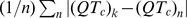 is the spatially averaged QT_c_ at a fixed measured position *k*, summed over the total number of nearest neighbors, *n*. For each channel, the average absolute difference between this value and the values of the neighboring channels was calculated. SI-QT_c_ was determined as the mean of these averaged differences over all channels. SI-QT_c_ increases with greater deviations in QT interval duration between neighboring registration sites. Second, the standard deviation among all the QT_c_ intervals in the QT_c_ contour map (SD-QT_c_) was calculated. Third, the spatial dispersion of QT_c_ interval (QT_c_ dispersion) was calculated as the difference between the longest and shortest QT_c_ interval in the QT_c_ contour map.

### Cardiac Magnetic Resonance (CMR)

All patients with TM were scanned with a 1.5-T magnetic resonance scanner (General Electric, HDx, Milwaukee, WI, USA) with multislice multiecho T2* approach, as previously described [22], [23]. A large and homogenous full-thickness region of interest was chosen in the left ventricular septum to assess myocardial T2*. Data analysis was performed using the commercial software (CMRtools, Cardiovascular Imaging Solutions). Cardiac T2* values less than 20 ms were considered abnormal [22], [24].

A continuous stack of short-axis cines was acquired to assess left ventricular end-diastolic and systolic volumes, masses, and ejection fractions, using standard techniques [25]. Data analysis was performed using a semiautomated edge detection program, as described previously [26], [27]. The ventricular volume and mass were indexed to the body surface area.

### Other Investigations

All participants recevied a 12-lead surface ECG before the MCG, to determine the QRS duration. For patients with TM, the following clinical and laboratory data were obtained from medical records and clincial evaluations at the time of study enrollment: hemoglobin and serum ferritin concentration (average value in the past 3 months before MCG), annual transfused-blood volume (cumulated value in the past 1 year before MCG), body weight and height, presence of diabetes mellitus, regimen of iron chelation therapy, and concurrent cardiac medications.

### History of Adverse Cardiac Events

Adverse cardiac events were defined as either heart failure at the time of study enrollment or significant arrhythmia, documented by 24-hour Holter ECG, which was performed every year on a regular basis. The diagnosis of heart failure was made if the patient complained of worsening dyspnea at rest or during exercise, a left ventricular ejection fraction below 56% (assessed by CMR), and if the treating physician made the clinical diagnosis of heart failure [28]. Clinically significant arrhythmias were categorized according to American Heart Association/American College of Cardiology guidelines [29], and included the following conditions: atrial fibrillation; atrial flutter; supraventricular or atrial tachycardia; ventricular fibrillation; ventricular tachycardia; potentially malignant ventricular premature complexes, which included ventricular couplet [30], multifoci ventricular premature complexes [31], and frequent unifocal ventricular premature complexes (≥30/h) [32]; significant bradycardia with sinus pause longer than 3 seconds [33]; and second-degree Morbitz type II or third-degree atrioventricular block [33].

### Statistical Analysis

Data are expressed as percentage, mean ± standard deviation, or median (25^th^–75^th^ percentile), as appropriate. Continuous variables were analyzed using the 2-sample *t* test or the Mann-Whitney U test, after testing for normality. Categorical variables were analyzed by the chi-squared test or Fisher's exact test, as appropriate. Linear relationships between variables were assessed after logarithmic transformation of T2* values using Pearson's correlation coefficient. Rreceiver-operating characteristic (ROC) curve analysis was conducted to test the diagnostic accuracy of indices of repolarization heterogeneity and cardiac T2* in relation to adverse cardiac events. Results were expressed in terms of the area under curve (AUC) and 95% confidence interval for this area. AUCs for various parameters were compared by the area test for correlated test results [34]. The best cut-off value was defined as the point with the highest sum of sensitivity and specificity. All data were analyzed using SPSS for Windows, version 13 (SPSS Inc, Chicago, Illinois, USA). A p value <0.05 was considered statistically significant.

## Results

### Subjects

The demographic, CMR, and clinical data of the 50 patients with TM are summarized in Table 1. Seven patients had diabetes mellitus requiring insulin therapy. None had a history of coronary artery disease or systemic hypertension. All patients were receiving iron-chelation therapy. Of the 50 patients, 41 patients (82%) were taking deferasirox, 5 patients (10%) were receiving deferoxamine combined with deferiprone, and the remaining 4 patients were receiving deferoxamine alone, deferiprone alone, deferoxamine plus deferasirox, and deferasirox plus deferiprone, respectively.

**Table 1:** Patient characteristics.

	Mean±standard deviation or median (25^th^–75^th^ percentile)
Age (years)	24.5±6.1
Male sex n (%)	24 (48)
Body mass index (kg/m^2^)	20.2±2.9
History of diabetes mellitus n (%)	7 (14)
Systolic/diastolic blood pressure (mmHg)	101±12/65±9
Transfusional blood volume (mL/year)*	13250 (11500–16250)
Pretransfusion hemoglobin (g/dL)†	10.2±1.2
Serum ferritin (ng/mL)	2002 (1357–3726)
CMR measures	
Cardiac T2* (ms)	27.9 (10.1–40.2)
LV end-diastolic volume index (mL/m^2^)	84.4 (75.4–100.1)
LV end-systolic volume index (mL/m^2^)	26.0 (20.8–32.3)
LV ejection fraction (%)	70.0 (64.0–73.0)
LV mass index (g/m^2^)	72.8 (65.6–84.2)
Adverse cardiac events n (%)	10 (20)
Heart failure n (%)	4 (8)
Arrhythmia n (%)	9 (18)
Atrial tachycardia	6
Atrial fibrillation and flutter	1
Combined ventricular and atrial tachycardia	1
Second-degree Morbitz type II AV block	1

* n = 33;

† n = 47.

AV = atrioventricular; CMR = cardiac magnetic resonance; LV = left ventricular.

The median cardiac T2* was 27.9 ms in our study patients. Twenty patients (40%) had abnormal cardiac T2* values (<20 ms). The left ventricular-ejection fraction was preserved in our study cohort. No difference in CMR-derived left ventricular measurements was apparent between patients with or without abnormal cardiac T2* values.

A total of 10 patients (20%) experienced adverse cardiac events at the time of study enrollment. Four patients had heart failure (all were in New York Heart Association functional class II), and all were receiving medical control (including diuretics, angiotensin-converting enzyme inhibitors, and/or beta-blockers). Nine patients had clinically significant arrhythmia (3 of them also had heart failure) documented by 24-hour Holter monitoring. Of those 9 patients, 6 had short-run atrial tachycardia, 1 had paroxysmal atrial fibrillation and flutter, 1 had short-run atrial and ventricular tachycardia, and 1 had second-degree Morbitz type II atrioventrilar block. Overall, patients experienced adverse cardiac events had significantly lower T2* (11.2±10.2 versus 31.6±16.3 ms, p<0.001) and higher left ventricular mass index (91.2±24.4 versus 74.8±19.3 g/m^2^, p = 0.029) comparing to those without. However, there are no differences in left ventricular volumes or ejection fraction between these 2 patient groups. In addition, despite all those 4 patients with heart failure had severely depressed T2* (ranged from 3.6 to 9.9 ms), they still exhibited preserved left ventricular ejection fraction (ranged from 55 to 73%).

### Spatial Repolarization Heterogeneity

MCG-derived parameters in both patients and healthy control subjects are shown in Table 2. Patients and control subjects were statistically alike in age and sex (age: 24.5±6.1 versus 25.4±3.8 years; male: 48% versus 49%), and the QRS duration was similar between these 2 groups (89±10 versus 91±11 ms). Compared to the healthy control subjects, patients with TM had not only a longer mean QT_c_ interval (p = 0.006), but also significantly greater values of SI-QT_c_, SD-QT_c_, and QT_c_ dispersion (all p values<0.001).

**Table 2:** Magnetocardiography-derived parameters in patients with patients with thalassemia major and control subjects.

	Patients (n = 50)	Controls (n = 55)	p
Mean QT_c_ interval (ms)	396±30	382±22	0.006
SI-QT_c_	9.8±2.5	7.3±1.4	<0.001
SD-QT_c_ (ms)	20.8±5.6	15.5±3.4	<0.001
QT_c_ dispersion (ms)	91.3±16.0	77.8±10.0	<0.001

QT_c_ = corrected QT interval; SI-QT_c_ = smooth index of corrected QT intervals; SD-QT_c_ = standard deviation of corrected QT intervals.

**Figure 2:**
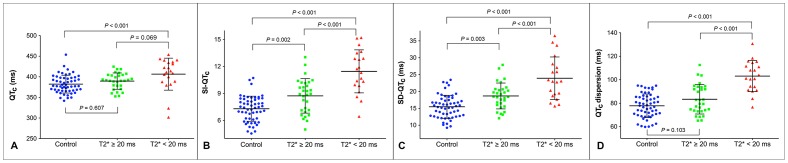
Comparisons of mean QT_c_ interval (A), SI-QT_c_ (B), SD-QT_c_ (C), and QT_c_ dispersion (D) in patients and controls. Patients were further divided into those with normal cardiac T2* value (≥20 ms) and those with abnormal cardiac T2* value (<20 ms). QT_c_ = corrected QT interval; SI-QT_c_ = smooth index of corrected QT intervals; SD-QT_c_ = standard deviation of corrected QT intervals.

Further analysis revealed an overall tendency for spatial repolarization heterogeneity to be low in the healthy control subjects, higher in patients with normal cardiac T2*, and highest in patients with abnormal cardiac T2* (Fig. 2). SI-QT_c_, SD-QT_c_, and QT_c_ dispersion all differed significantly between patients with and without abnormal cardiac T2* (all p values<0.001), and differed even more between patients with abnormal cardiac T2* and healthy controls (all p values<0.001). In addition, patients with abnormal cardiac T2* had greater SI-QT_c_ and SD-QT_c_, compared to the control subjects. However, for mean QT_c_ interval and QT_c_ dispersion, the group differences between patients with normal cardiac T2* and healthy controls were minimal.

### Analysis of Associations

**Figure 3:**
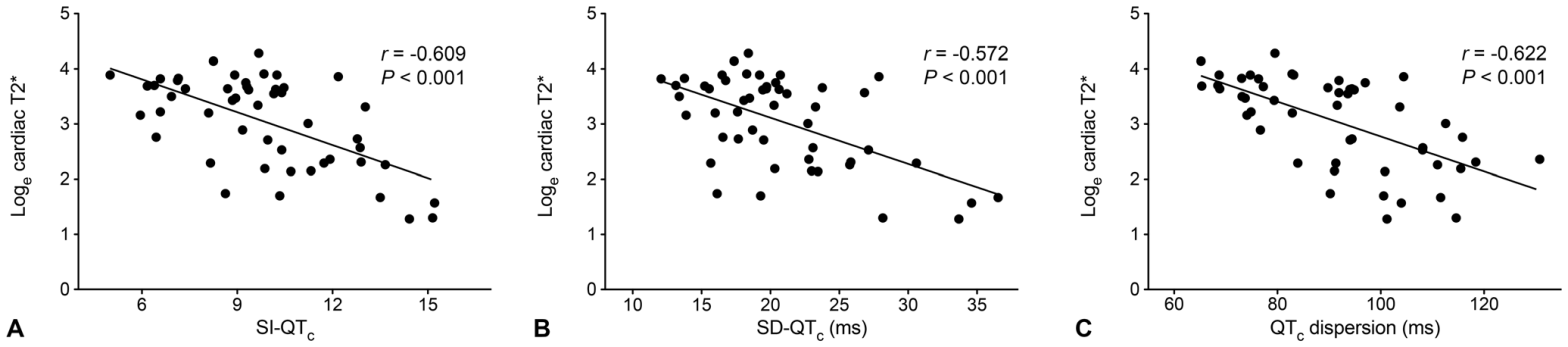
Correlations between SI-QT_c_ (A), SD-QT_c_ (B), QT_c_ dispersion (C) and log_e_ cardiac T2* value. QT_c_ = corrected QT interval; SI-QT_c_ = smooth index of corrected QT intervals; SD-QT_c_ = standard deviation of corrected QT intervals.

We observed directly negative correlations between log_e_ cardiac T2* value and SI-QT_c_ (r = −0.609, p<0.001), SD-QT_c_ (r = −0.572, p<0.001), and QT_c_ dispersion (r = −0.622, p<0.001) (Fig. 3), suggesting that spatial repolarization heterogeneity was related to myocardial iron load in patients with TM. Mean QT_c_ interval was also weakly related to cardiac T2* (r = −0.356, p = 0.011). No correlations were found between all 3 indices of spatial repolarization heterogeneity and QRS duration, left ventricular dimensions, ejection fraction, mass index, age, hemoglobin, and serum ferritin level, either in the overall study patients or in those with adverse cardiac events.

### Analysis of Receiver Operating Characteristic Curves for Adverse Cardiac Events

**Figure 4:**
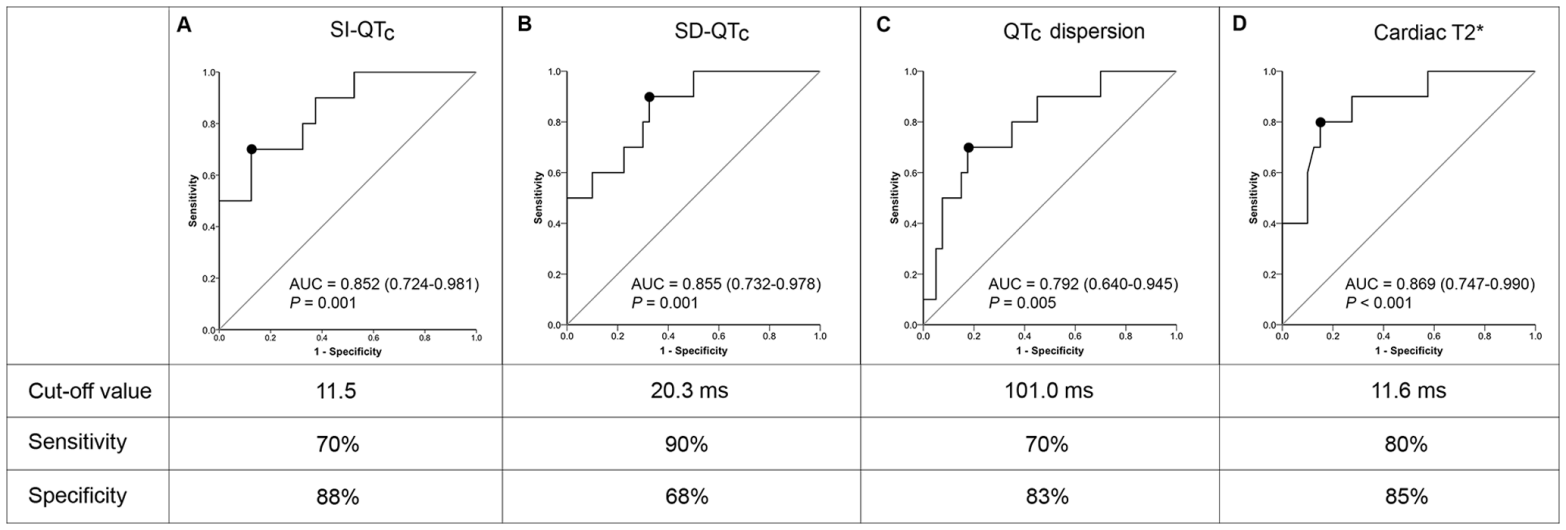
ROC curves of SI-QT_c_ (A), SD-QT_c_ (B), QT_c_ dispersion (C), and cardiac T2* (D) for predicting the presence of adverse cardiac events. The optimal cut-off value (labeled as the black dot on the ROC curve), sensitivity, and specificity of each individual parameter are shown. Data in the parenthesis indicate 95% confidence interval of the area under curve (AUC). QT_c_ = corrected QT interval; SI-QT_c_ = smooth index of corrected QT intervals; SD-QT_c_ = standard deviation of corrected QT intervals.

The ROC curves showed the overall performance of indices of spatial repolarization heterogeneity and cardiac T2* value for predicting the presence of adverse cardiac events (either heart failure or arrhythmia) (Fig. 4). AUCs for all 3 indices and cardiac T2* were all significantly larger than 0.5. We found no statistical difference between any 2 ROC curves. The cut-off value, as well as the sensitivity and specificity of each of these 4 values for predicting the occurrence of adverse cardiac events, are presented in Fig. 4. The cut-off values of SI-QT_c_ and QT_c_ dispersion were higher than the 95^th^ percentile of the data from control subjects, and the cut-off value of SD-QT_c_ was higher than the 90^th^ percentile of the data from control subjects.

We also found that left ventricular mass index significantly correlated with the presence of adverse cardiac events (AUC: 0.749, p = 0.017). In contrast, QRS duration, mean QT_c_ interval, other measurements of left ventricular function, hemoglobin, and serum ferritin level were not related to adverse cardiac events using the ROC curve analysis.

## Discussion

This study yielded 3 novel findings: (1) patients with TM had significantly greater spatial repolarization heterogeneity, compared to the healthy subjects; (2) the extent of spatial repolarization heterogeneity detected by MCG correlated with the severity of myocardial iron overload; and (3) spatial repolarization heterogeneity was linked to the presence of either heart failure or arrhythmia. Through the quantification of repolarization heterogeneity, our study results not only demonstrated the potential usefulness of multichannel MCG in the evaluation of TM patients who are at risk for cardiac iron overload, but also highlighted the importance of spatial repolarization heterogeneity in the pathophysiology of iron overload-related cardiac complications.

Increased cardiac repolarization heterogeneity has been noted in patients with TM [10]–[12], [35]. However, only a few studies investigated spatial repolarization heterogeneity [10], [12]. Rather than analyzing inter-lead variability of QT and JT intervals on the 12-lead ECG tracing used in the previous reports [10], [12], the present study used a multichannel MCG to detect signals from various locations of the heart, and provided more comprehensive measures of the spatial repolarization heterogeneity. We demonstrated that SI-QT_c_, SD-QT_c_, and QT_c_ dispersion were all significantly increased in this young cohort of TM patients, whose ventricular geometry and contractility were generally preserved. There was no direct correlation between repolarization heterogeneity and ejection fraction, even in ambulatory heart failure patients and those with clinical arrhythmias. Although the measurements of left ventricular function may change over time in the progression of cardiac dysfunction in TM patients, these measurements could only identify patients with an advanced stage of heart failure [36]. In addition, early ventricular dysfunction can be masked by supranormal cardiac function in response to chronic anemia [37]. Our study suggests that abnormal myocardial repolarization might be present early in the process of cardiac iron overload, when no evidence of overt ventricular dysfunction is visible.

Several mechanisms might be responsible for increased spatial repolarization heterogeneity in patients with TM. At the cellular level, iron can alter calcium homeostasis, which in turn affects cardiac action potential [38]. In addition, intracellular iron impairs the function of delayed-rectifier potassium channels [7]. Therefore, both calcium- and potassium-channel modification, caused by excessive cardiac iron, may result in repolarization abnormalities in patients with TM. At the tissue level, cardiac iron deposition is heterogeneous. Iron deposition is greater in the left than in the right ventricular myocardium, greater in ventricular free walls and septa than in atrial walls, greater in the subepicardial region than in the subendocardial region, and variable among various left ventricular regions [39]–[44]. These features might contribute not only to longer mean QT_c_ duration in TM patients, but also greater values of all 3 indices of spatial repolarization heterogeneity compared to those of the healthy subjects.

A close relationship between spatial repolarization heterogeneity and cardiac iron load indicated that increased repolarization heterogeneity is not just an intrinsic phenomenon in TM patients, who exhibit unique cardiac physiology related to mild chronic anemia [45]. All indices of spatial repolarization heterogeneity were not only greater in patients with significant iron overload, but also directly related to cardiac T2* values. This is the novel finding of the present study. A recent study pointed out that cardiac T2* heterogeneity increased markedly in patients with iron overload [43]. We therefore deduced that increased spatial repolarization heterogeneity in TM patients is attributed to greater heterogeneity in myocardial iron distribution, which is more pronounced in patients with cardiac iron overload. In contrast, a previous study by Ulger et al. [10] found that spatial repolarization heterogeneity (using QT_c_ dispersion on 12-lead surface ECG) positively correlated with left ventricular mass index (estimated by echocardiography), suggesting that spatial repolarization heterogeneity may be influenced by changes in left ventricular geometry. However, we could not find correlations between any of the 3 indices of repolarization heterogeneity and left ventricular mass index in our study cohort. We speculated that the discrepancy between our study results and that reported by Ulger et al. might be related to methodological differences in assessing repolarization heterogeneity and left ventricular mass.

Previous studies have shown that spatial repolarization heterogeneity is linked to arrhythmia and sudden cardiac death in patients with myocardial infarction, heart failure, and long QT syndrome [46]–[49]. However, little is known about the role of repolarization heterogeneity in relation to adverse cardiac events in TM patients. Our present study not only demonstrated a close relationship between spatial repolarization heterogeneity and cardiac T2*, but also provided evidence supporting the relationship between spatial repolarization heterogeneity, heart failure and arrhythmia. Currently, cardiac T2* may be the most powerful predictor for new-onset heart failure and arrhythmia in patients with TM [28]. Limited by the cross-sectional study design of our present study, we did not investigate the predictive value of these indices of spatial repolarization heterogeneity for the subsequent development of adverse cardiac events. However, based on comparisons among the ROC curves, these repolarization heterogeneity indices were at least equally accurate with cardiac T2* in distinguishing patients with and without adverse cardiac events at the time of study. Although ventricular repolarization heterogeneity was linked to adverse cardiac events (mostly are arrhythmias), the majority of arrhythmias originated from the atrium, but not from the ventricle. This finding is similar to that reported by Kirk et al [28]. One possible explanation for this is that the atrial myocardium is even more vulnerable to iron overload than the ventricular myocardium. Thus, greater ventricular repolarization heterogeneity caused by iron overload may serve as a marker for higher iron deposition in the atria. However, it remains technically difficult to directly measure cardiac T2* on the thin atrial myocardium. Another explanation is that atrial arrhythmia may reflect the overall hemodynamic burden placed on both the ventricles and atria. Further hemodynamic data are required to investigate this issue.

Of the 3 spatial repolarization indices used in this study, SD-QT_c_ and QT_c_ dispersion reflected global repolarization heterogeneity, and SI-QT_c_ reflected regional repolarization heterogeneity. Considering the associations with adverse cardiac events, all 3 indices exhibited similar performances. It is possible that both global and regional repolarization heterogeneity are pivotal in the development of cardiac complications related to iron overload. In addition, the cut-off value of each index of repolarization heterogeneity enabled clear separation of patients with TM from healthy subjects. Thus, the clinical use of repolarization heterogeneity detection by MCG in TM patients appeared to be justified.

As a noninvasive, contactless diagnostic tool, MCG could provide high spatial resolution to detect the imperceptible changes in cardiac electrical properties caused by various heart diseases in adults [13]–[18], [50] or fetal cardiac activity [51], [52]. Unfortunately, its availability remains quite limited in many countries, mostly attributed to the cost and set-up requirement. Furthermore, its superiority over other well-established imaging modalities remains to be determined. Therefore, for many physicians, MCG is still considered to be at most an interesting matter for research instrument so far. Future researches are mandatory to validate its usefulness in the clinical setting, as well as the potential application in pediatric population.

Our present study was limited by a cross-sectional study design, and therefore the predictive role of repolarization heterogeneity indices for subsequent occurrences of adverse cardiac events could not be investigated. Studies with a longer period of observation and, thus, a higher number of cardiac events, are required to validate the findings of this study. As the size of the population was not sufficiently large, the novel results of the present study must be considered as preliminary. The sensitivity and specificity of each cut-off value should be tested in further studies.

### Conclusions

Patients with TM exhibited significant changes in spatial repolarization heterogeneity, as detected by multichannel MCG. SI-QT_c_, SD-QT_c_, and QT_c_ dispersion are all related to myocardial iron load, and mark the presence of adverse cardiac events. Prospective studies are necessary to confirm the relationship between increased spatial repolarization heterogeneity and subsequent cardiac events in TM patients.
